# “Black People Like Me”: A virtual conference series to engage underserved patients with asthma in patient centered outcomes research

**DOI:** 10.1186/s40900-023-00428-3

**Published:** 2023-03-24

**Authors:** LeRoy Graham, Mary Hart, Michael Stinson, Rhoda Moise, Lynda Mitchell, Tonya A. Winders, Donna D. Gardner

**Affiliations:** 1 Allergy and Asthma Network, 10304 Eaton Place, Suite 100, Fairfax, VA 22030 USA; 2 East Point First Mallalieu UMC, East Point, GA USA; 3 Rhoda Moise, LLC (dba Dr. Rho Wellness), Philadelphia, PA USA

**Keywords:** Asthma, Disparities, Inequalities, Virtual, COVID-19, Patient-centered outcomes research

## Abstract

**Background:**

In response to racial inequity in asthma, asthma-related research among diverse patients is vital. However, people from historically marginalized groups are underrepresented in clinical and patient-centered outcomes research (PCOR). The “Black People Like Me” (BPLM) virtual conference series was developed to: (1) engage Black patients with asthma and their caregivers in education and discussions about asthma, and (2) encourage involvement in PCOR. Education about COVID-19 and COVID-19 vaccination was also incorporated.

**Methods:**

The Project Advisory Group consisting of Black patients, clergy, physicians, and a program evaluator met monthly to develop BPLM. The program consisted of free one-hour virtual sessions held monthly for 6 months. BPLM was promoted through the Allergy & Asthma Network website, emails, social media, and personal contacts with a recruitment goal of ≥ 100 Black patients with asthma or caregivers. Program evaluations, interactive polling questions during each session, and participant pre- and post-session tests were conducted.

**Results:**

Sessions averaged 658 participants including Black patients, family members, caregivers, Black clergy, health care providers, and other concerned community. Overall, 77% of participants strongly agreed with satisfaction with the sessions. Pre- and post-tests demonstrated that participants exhibited growth in knowledge regarding asthma risk, PCOR, and PCOR research opportunities for patients, exhibited preexisting and sustained knowledge regarding COVID-19 vaccination and side effects, and demonstrated an increased sense of empowerment during healthcare visits.

**Conclusions:**

BPLM demonstrated that a virtual platform can successfully engage Black communities. Incorporating clergy and religious organizations was critical in developing the trust of the Black community towards BPLM.

## Abstract (plainenglishlanguage)

There are more Black people with asthma in the US than White people. Black people have more hospital stays or deaths from asthma than White people. This highlights the need for Black people to be involved in research. Black people are missing from research. Patient centered outcomes research (PCOR) looks at patient treatment results. A webinar series titled “Black People Like Me” (BPLM) was developed for the Black community. This was a discussion about asthma, COVID-19, and PCOR between patients and experts. BPLM was a free, one-hour, virtual meeting held once a month for 6 months. BPLM was developed by Black patients, clergy, physicians, and a program evaluator. The goal was to meet the needs of the Black community. An average of 658 people participated in each virtual meeting. BPLM found that 77% of attendees were highly satisfied with the sessions. BPLM increased attendee knowledge of asthma, COVID-19, and PCOR. Attendees reported an increased willingness to be involved in PCOR. Including Black clergy helped BPLM be successful by encouraging trust in the program within the Black community. Future programs like BPLM may be developed to help people make decisions about being involved in their health care and PCOR.

## Background

Asthma poses a considerable burden on patients and the health care system. There is inequity in asthma prevalence in the US between individuals who identify as Black compared with those who identify as White (10.4% vs 7.9%, respectively) [1]. In addition, Black individuals are approximately 3-times more likely to die from asthma-related causes or visit an emergency department for asthma, and almost 4-times more likely be admitted to the hospital for asthma compared with White individuals [2].

Asthma is a heterogenous disease, and related research among diverse patient cohorts is vital. Patient populations and outcomes investigated in randomized controlled trials may not reflect real-world situations [3–5]. Patient-centered outcomes research (PCOR) can fill in some of the gaps associated with clinical trials by comparing outcomes relevant to daily life among many different treatment options and among diverse populations [5, 6]. Importantly, PCOR explores having the patient involved in different roles of research—individuals are not just a subject but a research partner [7].

People from historically marginalized groups are underrepresented in clinical trial research and PCOR [8–10]. Barriers to participation in clinical trials for people in historically marginalized groups include mistrust, lack of comfort with the clinical trial process, lack of information about clinical trials, time and resource constraints associated with trial participation, and lack of clinical trial awareness [11]. To significantly impact inequities in asthma outcomes, effective efforts to increase participation levels of patients from historically marginalized groups in research need to include patient engagement programs that address the many barriers. “Not One More Life” (NOML) is a non-profit program driven by health care volunteers and church leaders that engages historically marginalized communities in education, health screening, and health care related to asthma [12]. Based on learnings from NOML, the “Black People Like Me” (BPLM) virtual conference series was developed to: (1) engage Black patients living with asthma and their caregivers in education and discussions about asthma, and (2) encourage involvement in PCOR. With the start of the COVID-19 pandemic, a need was also seen to educate the Black community about the virus and COVID-19 vaccination, which was therefore incorporated into the BPLM series. A critical feature of the BPLM program is that it incorporated clergy and religious organizations to help develop trust in the Black community, based on learnings from NOML. Involvement of religious organizations had the added benefit of leveraging existing assets to facilitate meaningful engagement and discussion with the community.

## Methods

### Program development

Funding for the development of the BPLM conference series was through a Eugene Washington PCORI Engagement Award received by the Allergy & Asthma Network (AAN) from the Patient-Centered Outcomes Research Institute (PCORI). AAN is a non-profit patient education and advocacy organization for people with asthma, allergies, and related conditions. NOML currently operates as a program of AAN.

**Fig. 1:**
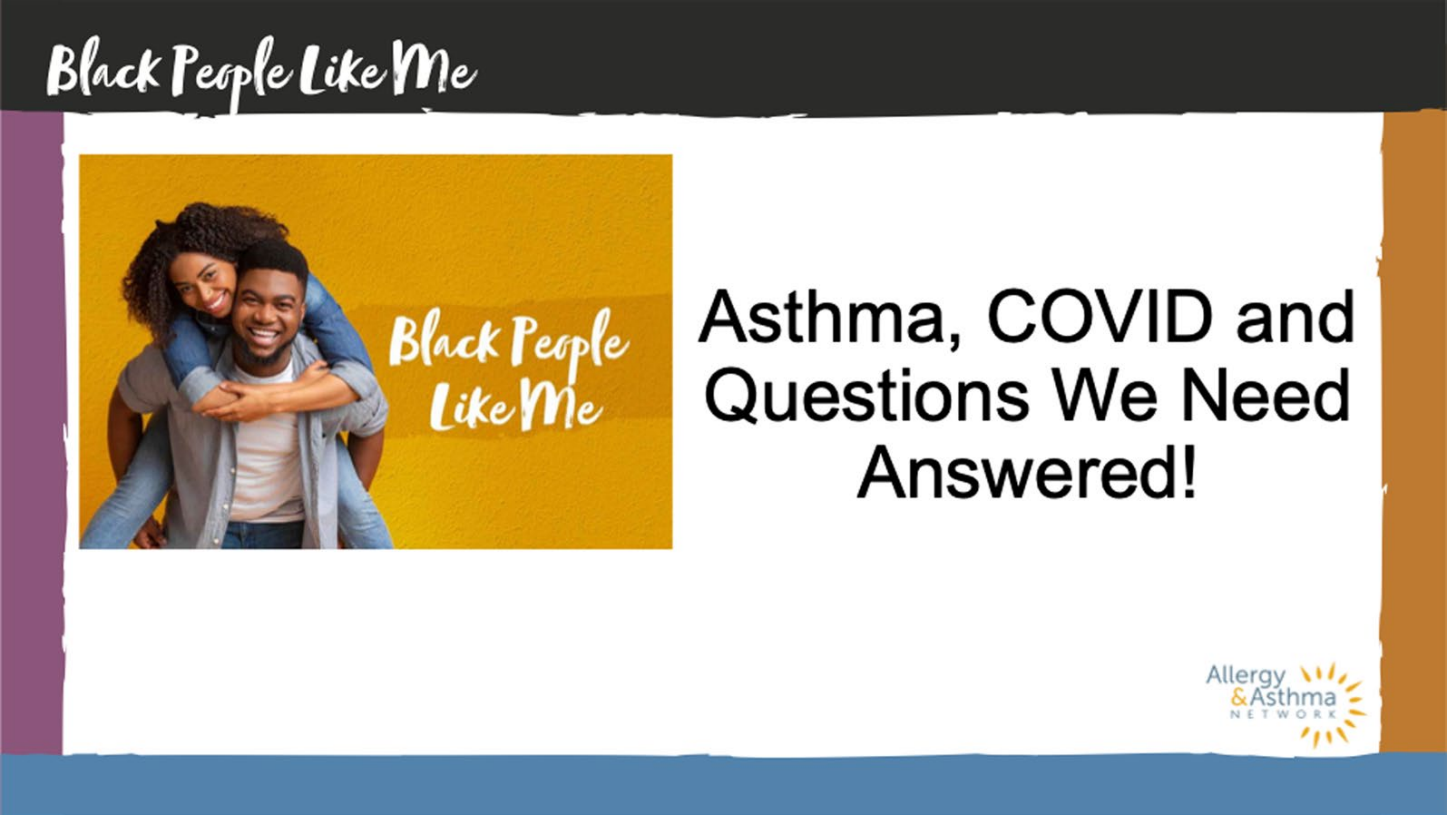
“Black People Like Me” virtual conference session Powerpoint template example

The first step in the BPLM conference series development was the establishment of a Project Advisory Group (PAG). The PAG consisted of Black patients, clergy, physicians with experience in PCOR and COVID-19, and a PCORI-experienced program evaluator. There was also a Project Lead (Dr. LeRoy Graham) and a Project Manager (Mary Hart). Meetings of the PAG occurred monthly where they recommended and discussed appropriately worded titles, session objectives, recruitment strategies, proposed images, suggestions for speakers, and topics of interest to the Black community. The conference platform, dates, times, and participant honorariums were all selected by the PAG to best meet the needs and expectations of the Black community. A title for the conference series of “Black People Like Me” was developed by the PAG, with a corresponding branded Powerpoint template created by the PAG and AAN. Colors, logo, and images used were chosen by the PAG to look like “Black People Like Me” and be diverse in sex, age, and community (Fig. 1).

### Virtual conference sessions

BPLM was a six-month series of one-hour virtual sessions held each month (Table 1). Registration for the conference was free. A virtual conference was necessary as the BPLM program was developed during the COVID-19 pandemic. The virtual format had the additional advantage of being less expensive than hosting an in-person conference. Zoom™ was chosen as the virtual platform because that was the platform the Black community was most comfortable with using to stay connected and attend religious services during COVID-19–related lockdown and social distancing procedures. In addition, Zoom™ could be used from a smartphone and did not require installation of an app.

**Table 1:** “Black People Like Me” virtual conference session titles and attendance

Month, session no	Topic	Meeting duration (Minutes)	Registered participants	Attendance N (Rate %)	Question and answer, N
December, Session 1	Asthma, COVID-19, Questions We Need Answered: Black People Like Me Series	108	N = 1412	Patient/family, n = 113	N = 24
				HCP, n = 377	
				Clergy, n = 9	
				Unknown, n = 81	
				Total, 580 (41%)	
January, Session 2	African Americans and Research: Making it Work for Folks Like Us	89	N = 960	Patient/family, n = 176	N = 26
				HCP, n = 225	
				Clergy, n = 6	
				Unknown, n = 84	
				Total, 491 (51%)	
February, Session 3	COVID-19 and Black Folk: Changing the Game, Changing the Outcome	103	N = 1006	Patient/family, n = 217	N = 37
				HCP, n = 203	
				Clergy, n = 11	
				Unknown, n = 80	
				Total, 511 (51%)	
March, Session 4	Asthma and COVID-19: Is Research Really that Important To Me?	79	N = 812	Patient/family, n = 182	N = 18
				HCP, n = 105	
				Clergy, n = 8	
				Unknown, n = 238	
				Total, 533 (66%)	
April, Session 5	Asthma and COVID-19: My Journey with Asthma, COVID or Other Health Issues…Letting Researchers Know What Questions We Need Answered	96	N = 1733	Patient/family, n = 560	N = 58
				HCP, n = 60	
				Clergy, n = 12	
				Unknown, n = 415	
				Total, 1047 (60%)	
May, Session 6	Patient Advocacy in Asthma & COVID-19: Where Do We Go from Here?	105	N = 1668	Patient/family, n = 616	N = 41
				HCP, n = 61	
				Clergy, n = 18	
				Unknown, n = 88	
				Total, 783 (47%)	
Average		100	N = 1265	658 (52%)	N = 34

*HCP* health care provider

Information from the designated speaker was transferred to the BPLM template and polling questions about the topic being discussed were inserted in the presentation to gain immediate feedback from the participants. The format for each session included a welcome from the program moderator and Project Lead, and an introduction of the topic, agenda, and objectives. Speakers presented their topic(s) while interactive polling questions, chat, and Q&A took place throughout the session. The Project Manager or AAN staff communicated the questions from the chat and Q&A to the moderator to ask the speakers and panelists. AAN support staff used Twitter (#BlackPeopleLikeMe) to send out real-time messages throughout each session. All sessions were recorded and uploaded to the AAN website at: http://blackpeoplelikeme.com/.

Patient Advisor speakers for the session were from diverse backgrounds and were allowed to tell their stories from their experience, from their heart. No prepared scripts were used. Practice sessions with the moderator and all speakers were held a few days before the conference sessions to ensure everyone was familiar with the platform, camera and microphone use, program flow, polling questions, and Q&A, and go over the backup plan if anyone lost connection, etc.

To further engage the community and maintain momentum between sessions, a #BlackPeopleLikeMe Facebook group was created and managed by AAN. Session participants were encouraged to join and those who did were asked to share their experiences living with asthma, COVID-19, and related conditions by posting images, quotes, or poems to describe their feelings and journey this past year.

### Recruitment

The recruitment goal for BPLM was to engage at least 100 Black patients with asthma or caregivers of patients with asthma. The general public was also invited. AAN posted the event on their website, sent emails to 40,240 patients, families, and health care providers, posted Twitter tweets, and advertised the sessions in all their social media groups (e.g., LinkedIn, Instagram, and Facebook). In addition, PAG members recruited participants through personal emails, phone calls, and by contacting clergy and physician offices. Participation incentives were provided at the end of each session in the form of an electronic emailed gift card to the first 100 attendees from the US who indicated they were patients with asthma and who stayed for the entire session.

### Program evaluations

Program evaluations included both quantitative and qualitative methods to comprehensively assess impact. Quantitative methods included written evaluations that were administered after every session to rate the overall satisfaction of program materials, speakers, topics, and use of virtual program format for registration and live virtual sessions. In addition, interactive polling questions related to the session topic were administered during each session. Participants also took a pre- and post-session test to assess knowledge of COVID-19, health risk for people with asthma and related symptoms, health inequity, disease management, preventative measures, and research engagement. The links to the pre- and post-tests were emailed to participants when they registered for the program and after the program. Qualitative evaluations included assessment of the Q&A portion of each session and comments posted in the chat box.

## Results

### Session participation and responses

Session participants included Black patients, family members, or caregivers, Black clergy, health care providers, and other concerned community from across the US, with some non-US participants. The sessions averaged 658 participants with an average duration of approximately an hour and 40 min each (Table 1). Sessions ran over the allotted 1 h because participants remained engaged by submitting comments and questions for discussion.

Program evaluations found that overall, 77% of participants strongly agreed with satisfaction with various aspects of the sessions (Table 2). Participants mainly shared positive feedback highlighting their delight in the interactivity of the sessions, educational and informational enhancement opportunities, inclusion of both expert physicians and researchers, and experience from community members and patients. Participants desired more engagement through handouts and shared access to other outlets such as church groups. Additionally, comments included room for improvement with technology and sensitivity around race and time. Overall, the chat box themes highlighted the safe space created with the BPLM program along with participants’ appreciation for the information and education shared. Participant questions submitted during the Q&A portion centered around the COVID-19 vaccine, disease management, health equity, and outreach. Sample questions from the sessions are shown in Table 3.

**Table 2:** “Black People Like Me” virtual conference session evaluation results

Evaluation question	Answer Choices	December N = 178* Responses N (%)	January N = 183* Responses N (%)	February N = 168* Responses N (%)	March N = 144* Responses N (%)	April N = 319* Responses N (%)	May N = 233* Responses N (%)
It was easy to register for the program	Strongly Agree	159 (90%)	154 (84%)	151 (90%)	120 (83%)	240 (75%)	173 (74%)
	Agree	17 (10%)	28 (15%)	17 (10%)	23 (16%)	76 (24%)	57 (24%)
	Disagree	1 (1%)	–	–	–	3 (1%)	1 (1%)
	Strongly Disagree	–	1 (1%)	–	1 (1%)	–	2 (1%)
I felt encouraged and safe to participate during the session	Strongly Agree	140 (79%)	138 (76%)	134 (78%)	115 (80%)	242 (77%)	176 (77%)
	Agree	37 (21%)	42 (23%)	34 (20%)	29 (20%)	70 (22%)	52 (23%)
	Disagree	–	–	–	–	1 (1%)	–
	Strongly Disagree	–	2 (1%)	–	–	2 (1%)	2 (1%)
The speakers were experts and taught me a lot about COVID and Asthma	Strongly Agree	126 (71%)	111 (61%)	133 (80%)	105 (73%)	239 (76%)	185 (79%)
	Agree	47 (26%)	66 (36%)	32 (19%)	38 (26%)	74 (23%)	48 (21%)
	Disagree	5 (3%)	5 (3%)	2 (1%)	1 (1%)	2 (1%)	–
	Strongly Disagree	–	–	–	–	1 (1%)	1 (1%)
I was satisfied with the topics presented	Strongly Agree	134 (75%)	123 (68%)	129 (78%)	108 (76%)	227 (72%)	178 (76%)
	Agree	41 (23%)	57 (31%)	36 (22%)	31 (22%)	86 (27%)	54 (23%)
	Disagree	3 (2%)	1 (1%)	1 (1%)	2 (1%)	2 (1%)	–
	Strongly Disagree	–	1 (1%)	–	–	2 (1%)	1 (1%)
The Zoom Webinar was a good way to present this information	Strongly Agree	139 (78%)	133 (73%)	135 (80%)	106 (75%)	237 (75%)	164 (71%)
	Agree	39 (22%)	49 (27%)	33 (19%)	34 (25%)	79 (25%)	64 (28%)
	Disagree	–	–	–	–	2 (1%)	2 (1%)
	Strongly Disagree	–	1 (1%)	–	–	–	1 (1%)
I felt like the presenters answered the questions at the end	Strongly Agree	87 (50%)	114 (63%)	113 (68%)	98 (69%)	193 (62%)	149 (64%)
	Agree	75 (43%)	65 (36%)	49 (29%)	42 (30%)	110 (35%)	78 (34%)
	Disagree	11 (6%)	2 (1%)	5 (3%)	2 (1%)	9 (3%)	3 (1%)
	Strongly Disagree	–	–	–	–	1 (1%)	2 (1%)
I liked having the polling questions during the presentation	Strongly Agree	125 (71%)	137 (76%)	134 (80%)	99 (69%)	194 (61%)	166 (71%)
	Agree	51 (29%)	42 (23%)	33 (20%)	43 (30%)	116 (37%)	64 (27%)
	Disagree	–	2 (1%)	–	1 (1%)	6 (2%)	2 (1%)
	Strongly Disagree	–	–	–	–	–	1 (1%)
I was satisfied with the overall session	Strongly Agree	135 (77%)	135 (74%)	136 (81%)	108 (76%)	232 (73%)	167 (72%)
	Agree	40 (23%)	45 (25%)	31 (18%)	32 (22%)	82 (26%)	63 (27%)
	Disagree	1 (1%)	2 (1%)	1 (1%)	2 (1%)	2 (1%)	1 (1%)
	Strongly Disagree	–	1 (1%)	–	1 (1%)	–	1 (1%)

*Monthly totals may not be uniform due to percentile rounding to whole numbers and participants skipping questions

**Table 3:** Sample questions posed by participants during the “Black People Like Me” virtual conference sessions

Themes	Sub-themes	Sample responses
Vaccine	*Safety*	*What if you are allergic to aspirin? Is the vaccine safe?*
	*Dosage*	*Are there any repercussions to missing the second COVID-19 vaccine?*
	*Longevity*	*Will we need a booster vaccine in the fall?*
	*Access*	*Where can I get vaccinated?*
	*Efficacy*	*With the vaccine, can you still get and spread the virus?*
	*Side effects*	*What are the long-term effects of the vaccine? Infertility? Can the vaccine cause loss of taste, smell, or both?*
	*Hesitancy*	*How do you instill trust with Black Americans that the vaccine is safe?*
	*Allergy interactions*	*Those who are denied a vaccine due to allergies what option of defense due they have?*
	*Variants*	*If we've had the two vaccines, is it safe to join society again in light of the variants?*
Disease management	*Children*	*What’s the best way to help children with asthma make their lungs stronger (or make asthma less of a problem)? Exercise? Certain foods?*
	*Age*	*What is the lifespan of people living with asthma on medication vs without? How long do they live? What specific age groups are meant by "elderly" versus "younger"?*
	*Recommendations*	*It would be great if an app is developed to provide feedback regarding controller medication use to the provider and the parent, similar to diabetes type 1 care*
	*Interrelated factors*	*Is there any significant link between depression and asthma?* A*sthma and COPD, what is the difference and is there any link? Does stress trigger asthma? Is having asthma a risk factor in COVID-19? Is asthma a disability?*
	*Treatment options*	*Is there a long-term asthma treatment under development or testing that may change lives of the asthmatic community? Is asthma curable like malaria? Why is it important for asthma patients to stick to an asthma control action plan?*
Health equity	*Environment*	*What role does environmental racism play in the disproportionate numbers in Black Americans with asthma?*
	*Race and comorbidity*	*In the study with African Americans and Latinos with asthma, were comorbidities a factor in emergency room visits and asthma related deaths?*
	*Race*	*I've personally not taken the COVID-19 vaccine. Do you think we should take it, as Blacks, or we should abstain? I feel we may be given substandard ones. Why are Black people more affected by COVID-19 than White people?*
	*Sex*	*Why does COVID-19 tend to affect more men than females?*
Outreach	*Education*	*What are some ways that we can educate our communities about asthma?*
	*Webinar distribution*	*When will this webinar be available for rebroadcast? Will this session be archived so that we can share it with others who were not able to log on?*
	*Patient engagement*	*How can someone become a patient partner in a study?*

Pre- and post-tests conducted for session 1 helped the PAG with restructuring evaluations for future sessions. Highlights of the pre- and post-test results from sessions 2–6 are shown in Table 4. Session 2 pre- and post-tests demonstrated that participants exhibited growth in knowledge regarding asthma risk and research opportunities for patients, and in session 3, participants exhibited preexisting and sustained knowledge regarding COVID-19 vaccination procedures and side effects. For session 4, participants exhibited improvements regarding knowledge of PCOR, sustained awareness of PCORI training opportunities for patient involvement in research, and increased implementation of COVID-19 vaccination. Polling questions in session 4 had a greater number of responses than the pre- and post-tests and reflected an increased willingness by participants after the expert presentations to take the COVID-19 vaccine and to participate in clinical research. In session 5, participants demonstrated an increased sense of empowerment in reference to participants perceived knowledge in the engagement process and treatment decisions during health care visits. There was an increase in the perception that getting health care or treatment for a health issue is a problem for some Black communities (88% [n = 135/155] pre-test to 98% [n = 64/65] post-test). In the last session, participants exhibited an increased knowledge of COVID-19 long hauler health issues, higher rates of vaccination, and awareness of PCORI research opportunities. Participants also reported increased interest in serving as a patient advisor with AAN (25% [n = 27/117] pre-test to 31% [n = 17/68] post-test).

**Table 4:** “Black People Like Me” virtual conference session pre- and post-test result highlights

Month, session no	Assessment question	Answer choices	Pre-test Responses N (%)*	Post-test Responses N (%)*
January, Session 2			N = 71	N = 40
	“Asthma exacerbation” is when asthma symptoms get worse. You may need steroids or to go to the hospital	True	67 (94%)	39 (100%)
		False	4 (6%)	0
	The death rate for African Americans with Asthma compared to Caucasians is:	No difference	2 (3%)	0
		2 times less	7 (10%)	1 (3%)
		2–3 times greater	43 (61%)	31 (78%)
		10 times greater	19 (27%)	8 (20%)
	Patient Centered Outcomes Research (PCOR)	Is how research findings move into real world practice	13 (19%)	6 (15%)
		Helps people and their caregivers communicate and make informed decisions, allowing their voices to be heard in assessing the value of health care	41 (59%)	28 (70%)
		Research facts that make results true	0	4 (10%)
		The study of patient experience	16 (23%)	2 (5%)
	Asthma patients can participate as research advisors	True	68 (96%)	36 (90%)
		False	3 (4%)	4 (10%)
		I don’t know	0	0

February, Session 3			N = 93	N = 116
	Once I have taken the COVID-19 vaccine I no longer need to wear a mask or distance myself from others because I can’t catch COVID-19	True	2 (2%)	1 (1%)
		False	90 (98%)	115 (99%)
	It is important to take ________ doses of the Moderna COVID-19 vaccine for it to properly work? (choose only one)	1 dose	1 (1%)	1 (1%)
		2 doses	91 (98%)	115 (99%)
		3 doses	1 (1%)	0
		4 doses	0	0
	The most common side effects people are reporting from getting the COVID-19 vaccine are: (select all that apply)	Sore arm	80 (86%)	107 (92%)
		Flu like symptoms for 24–48 h	61 (66%)	81 (70%)
		Nose bleeds	0	1 (1%)
		Tiredness/fatigue	60 (65%)	84 (72%)

March, Session 4			N = 68	N = 38
	PCOR is an acronym for:	Project center on research	2 (3%)	0
		Patient-centered outcomes research	63 (93%)	36 (95%)
		Patient centers on research	3 (4%)	2 (5%)
		None of the above	0	0
	PCORI has training sessions/videos to help train patients to learn how they can be more involved in all stages of research	True	68 (100%)	38 (100%)
		False	0	0
	There is still hesitancy in the Black community to get the COVID-19 vaccine. Will you take it or have you taken it?	Yes, I will take it when it is available to me	33 (49%)	12 (32%)
		Yes, I have already taken it	23 (34%)	19 (50%)
		No, I will not take it	12 (18%)	7 (18%)

April, Session 5			N = 155	N = 65
	Who has the greatest knowledge in the engagement process?	Government officials, researchers	30 (19%)	5 (8%)
		Doctors, nurses	64 (41%)	17 (26%)
		Community – you	54 (35%)	41 (63%)
		I don’t know	7 (5%)	2 (3%)
	When you visit your doctor or health care provider, how do you generally decide what treatment or medicines you will take for your condition?	The doctor knows best about what I need and prescribes it	53 (34%)	9 (14%)
		The doctor explains all the options available to me, we discuss the options together and I make the decision based on what is right for me	82 (53%)	51 (79%)
		I request to take what my neighbor is taking because it is less expensive	3 (2%)	0
		The decision is based on what my insurance company will cover	13 (8%)	3 (5%)
		Other	3 (2%)	2 (3%)
	Is getting health care or treatment for a health issue a problem for some Black communities?	Yes	135 (88%)	64 (98%)
		No	12 (8%)	1 (2%)
		I don’t know	6 (4%)	0

May, Session 6			N = 117	N = 68
	What are some commonly reported lingering health issues from having COVID-19? (Choose all that apply)	Anxiety	60 (51%)	46 (68%)
		Fatigue	86 (74%)	56 (82%)
		Racing heartbeat	49 (42%)	42 (62%)
		Brain fog – problems with memory or concentration	51 (44%)	51 (75%)
		Shortness of breath	99 (85%)	57 (84%)
	Have you received the COVID-19 vaccine? (Choose only one)	Yes	79 (68%)	54 (81%)
		No	26 (22%)	7 (11%)
		No access to the vaccine where I live	6 (5%)	0
		Do not plan to take it	1 (1%)	1 (1%)
		Waiting for my second shot	5 (4%)	5 (7%)
	Any member of a community may participate in a PCORI research study or engagement award. (Choose only one)	True	92 (79%)	57 (84%)
		False	25 (21%)	11 (16%)
	What are some examples of how you would like to get “involved” after being a part of these sessions? (Choose all that apply)	Help out with Community asthma, COVID outreach/education	85 (77%)	38 (69%)
		Sign up to become an Allergy & Asthma Network Volunteer	41 (37%)	19 (35%)
		Sign up as an Allergy & Asthma Network Patient Advisor	27 (25%)	17 (31%)
		Join the Allergy & Asthma Network Asthma360 Research Registry	49 (45%)	17 (31%)
		Contact PCORI about becoming a PCORI Patient Advisor	25 (23%)	12 (22%)
		Sign up and participate in a Research focus group or study	62 (56%)	26 (47%)
		Contact Allergy & Asthma Network for more information about how to become more involved in helping others, telling my story, etc	43 (39%)	19 (35%)

*Monthly totals may not be uniform due to percentile rounding to whole numbers and participants skipping questions

### Social media engagement

Facebook received more than 12,000 impressions and an engagement of over 225 accounts during the BPLM series, Instagram received over 6900 impressions and an engagement of over 100 accounts during the BPLM series, and Twitter received over 53,000 impressions and engagement of over 580 accounts during the BPLM series. The private BPLM Facebook group currently has 132 members, and from March 2022-March 2023, 94 posts have been created, there have been 311 comments from members, and 566 reactions from members on the posts.

## Discussion

BPLM demonstrated that a virtual platform can be successful in engaging Black communities. The virtual conference far exceeded the goal of 100 participants. Clearly the community was curious about getting involved in research, and participants took time out of their day to attend because the sessions focused on questions that were important to them. Current, real-world conversations were held that had an impact on participant perceptions. One of the sessions was provided by a representative from PCORI that explained how and where to find more information about engaging as a patient partner in PCOR and clinical research. Many people had not considered their potential role as a partner in research, and this topic could be a focus for future sessions. Increasing patient involvement in research can identify new areas of concern or for health care improvement that may not be considered by health care providers and researchers [13]. The sessions also changed perceptions about how misinformed or uninformed participants were about COVID-19 and COVID-19 vaccines.

The intention of BPLM was to inform participants how they could become involved in PCOR. After the program, participants were added to a distribution list for the AAN research newsletter, and when opportunities to participate in research were identified, this information was sent to participants using the same distribution list. The BPLM Facebook group also posts information about research participation opportunities. To date there has been no follow-up to see if any participants of BPLM actually chose to become engaged in PCOR, and this may be a subject of future research.

There were some challenges to the BPLM program. Early on the patient advisors from the PAG were not as engaged as expected. The Project Lead and Manager held one-on-one meetings with each of the patient advisors and held a few monthly meetings with just the patient advisors to get their feedback about the project and to help promote more engagement in the group. Eventually the group was more vocal and bonded with time. Technical issues were the main challenge with the virtual sessions. The PAG learned from the first session that more direct and detailed instructions to presenters and panelists was needed in how to use the virtual platform. A speakers instruction list was created for the team to use with each session and the number of presentation and technical issues was reduced for the rest of the sessions.

There was a concern that the virtual session format would not keep the attention of the participants. Different types and styles of educating and communicating with the participants (e.g., patient storytelling, chat, interactive polling questions) were used to help keep participants from getting distracted when listening or watching a session online rather than in-person. However, the virtual format cannot replace the full personal approach of an in-person conference. Judging from the response and attendance with these virtual sessions, a hybrid of both virtual and in-person conferences may be the best approach for future conferences.

Gaining the trust of the community was a goal of BPLM. Research shows that compared with White individuals, Black individuals are more likely to trust health information from community stakeholders, such as religious organizations and charitable organizations [14]. Therefore, such organizations should be considered to assist in dissemination of health care information targeted toward the Black community. Using the principles from NOML, clergy assisted and led the way in engaging their community and building a foundation of trust in BPLM. In addition, the patient advisors in the PAG proved critical to creating genuine, honest, and relatable sessions that people responded to and were excited about throughout all six sessions.

## Conclusions

BPLM successfully engaged Black communities in the topics of asthma, COVID-19, and PCOR using a virtual platform. Incorporating clergy and religious organizations was critical in developing the trust of the Black community towards BPLM. It is our hope that BPLM will have far reaching and long-lasting effects in the Black community, such as increasing patient involvement in PCOR and increasing the sense of empowerment during an individual’s health care journey. We anticipate that learnings from BPLM will lead to future engaging programs.

## Data Availability

The datasets generated during and/or analyzed during the current study are available from the corresponding author on reasonable request.
